# Sibling Relation, Ethnic Prejudice, Direct and Indirect Contact: There is a Connection?

**DOI:** 10.5964/ejop.v11i4.958

**Published:** 2015-11-27

**Authors:** Sara Alfieri, Elena Marta

**Affiliations:** a Psychology Department, Università Cattolica del Sacro Cuore, Milan, Italy; University of Liverpool, Liverpool, United Kingdom

**Keywords:** sibling relation, ethnic prejudice, direct contact, indirect contact, socialization, family relation

## Abstract

The literature on the socialisation of prejudice has concentrated on “vertical” processes (from parents to children), ignoring siblings’ contribution. This work aims to investigate the effect of contact (direct or indirect) with the outgroup that young people experience a) directly or b) indirectly through older or younger siblings’ friendships. Our hypotheses are a) that young people with friends in the outgroup will report lower prejudice levels (direct contact), as will young people who have older or younger siblings with friends in the outgroup (indirect contact); b) that other forms of contact such as having classmates/coworkers, neighbours, or employees are not effective in reducing either direct or indirect prejudice. 88 sibling dyads were administered the blatant and subtle prejudice questionnaire (Pettigrew & Meertens, 1995) and some ad hoc items aimed at investigating the typology of the contact experienced. The analysis of mixed ANOVA reveals that the first hypothesis was partially confirmed in that prejudice (subtle for the younger sibling and blatant for the older one) decreases in a statistically significant way only when there is the co-presence of direct and indirect contact. The second hypothesis is fully confirmed as no statistically significant differences emerged between the groups.

The recent global economic crisis has accentuated migratory phenomena and, consequently, also the contacts between different ethnic groups. Often the encounter among outgroups is influenced by the prejudices of people from the host countries. Among the various sources for these biases there is also the family, which numerous research has demonstrated plays a fundamental role in this process, mainly for the younger generations (see for example Castelli, Carraro, Tomelleri, & Amari, 2007; Castelli, Zogmaister, & Tomelleri, 2009; O’Bryan, Fishbein, & Ritchey, 2004; Sinclair, Dunn, & Lowery, 2005). Usually, when it comes to family influence, however, the parents come first to mind. Even though the sibling relation is fundamental it has received little attention as regards processes of influence with respect to the theme of prejudice.

The present work intends to investigate the sibling relation’s role in reducing ethnic prejudice. In this article, we present research based on the known theory of direct contact hypothesis (Allport, 1954; Williams, 1947) and indirect contact (Wright, Aron, McLaughlin-Volpe, & Ropp, 1997) applied to the sibling relationship.

## Blatant and Subtle Prejudice

Prejudice is an unjustified feeling of dislike or hatred towards members of ethnic minority groups (Brown, 1995).

Pettigrew and Meertens (1995; Meertens & Pettigrew, 1997) differentiate between two correlated but distinguishable forms of prejudice: blatant and subtle. Blatant prejudice is “the traditional form; it is hot, close, and direct” (Meertens & Pettigrew, 1997, p. 54). This form is distinguished essentially by the threat perceived as regards to an external group and the resulting rejection of that group. The people characterized by higher levels of manifested prejudice perceive the outgroup as threatening or genetically inferior and this induces them to experience negative feelings (like fear, anger, and disgust) towards them and to avoid any contact with its members. Consequently, there is a refusal to have intimate contact (friendship, romantic, or sexual relations) with persons in the outgroup. This resistance reveals itself to be a strong element of power because not only is intimate contact rejected, but also any relationship that entails the superiority of the outgroup discriminated against (for example, leadership positions held by the minority) (Pettigrew & Meertens, 1995).

On the other hand “Subtle prejudice is cool, distant, and indirect” (Meertens & Pettigrew, 1997, p. 54). The distinctive features of subtle prejudice are coldness and aloofness expressed in such an indirect way as to be seen as socially acceptable and in full compliance with the norms of civil society, allowing the person to have a non-discriminating and prejudice free image of him/herself. This is actualized, for example, in the fierce defense of traditional values and of the status quo (or even of the superiority of the ingroup), the exaggeration of the intercultural differences, and the belief that the outgroup enjoys unmerited rights and benefits. In addition, the rejection of positive emotional responses toward the outgroup is also conspicuous. (Pettigrew & Meertens, 1995).

## The Contact Hypothesis

At the basis of the concept of prejudice is the idea that it is caused by limited acquaintance with the outgroup. It follows that increasing familiarity with a group can lead to greater acquisition of more correct opinions about it. The theory that has attempted to substantiate this supposition is the contact hypothesis by Williams (1947); it was taken up by Allport (1954) a few years later, and by many others after him (for a review, see Brown & Hewstone, 2005; Dixon, Durrheim, & Tredoux, 2005; Pettigrew & Tropp, 2006, 2008).

The efficacy of the contact hypothesis theory requires that the social groups satisfy the following conditions:

1. they promote a *similar status*: it is probable that contact between the members of two or more groups that differ in status and power reinforces, rather than decreases, prejudice and attitudes of superiority in the dominant group in that it reflects asymmetries already present in the social context;
2. they promote *in-depth interactions*: in order for the contact to be efficacious, it must be prolonged over time so as to allow for a deep, rather than superficial, acquaintance. Allport (1954) maintained that the critical point is not physical proximity: people who live next to a Chinese neighbourhood have occasion to see many people belonging to this ethnic group. Nevertheless, such contacts are superficial. This type of contact does not undermine prejudice: on the contrary, it seems to increase it in that clichés, traditions, and stereotypes connected to a given group can be more accessible;
3. it is required, on the part of the groups involved, that they achieve *common objectives*. If the contact leads the hostile groups’ members to have a superordinate goal, it is possible to produce a variation in attitude;

Looking at the family as a group, and in particular at the sibling relationship, it can be characterized by all the above conditions.

The efficacy of contact for reducing prejudice has emerged from numerous studies in different socio-cultural contexts, which can be differentiated as regards both the groups and social relations investigated as well as the methodologies used (Pettigrew & Tropp, 2006, 2008).

Pettigrew and Tropp (2006) through an important meta-analysis revealed that the relation between contact and prejudice is statistically significant and negative. Furthermore, they found that contact with friends belonging to an outgroup is more efficacious in reducing prejudice as compared to more generic types of contact.

In synthesis, therefore, the contact hypothesis is generally valid (for different positions, see Brown, 1995; Dovidio, Gaertner, & Kawakami, 2003). One of this theory’s limitations consists in the problem of the generalisation of positive experience: often more positive attitudes develop toward real participants in the contact situation, but very limited changes toward other outgroup members, in general, are seen (Hewstone, 1996; Miller, 2002).

## ‘Indirect’ Contact

Starting from the “classic” contact hypothesis, many extensions and variations of it have been investigated. One of the most widely investigated forms of contact is the ‘extended’ (*indirect*) form. Wright and colleagues (1997) proposed the concept of extended contact to indicate that even the mere fact of knowing that one or more members of one’s ingroup have friends in the outgroup can reduce prejudice. The principle is the following: if one of our friends can name in his circle of friends a person from the outgroup, whom we do not know directly, it is probable that we will not harbour prejudice toward this person but, rather, good will.

The efficacy of this type of contact was confirmed in other works (Cameron & Rutland, 2006; De Tezanos-Pinto, Bratt, & Brown, 2010; Turner, Hewstone, Voci, Paolini, & Christ, 2007; Wright, Aron, & Brody, 2008). The most interesting aspect of this work resides in the fact that the relation between extended or indirect contact and a reduction in prejudice is significant even when there is no direct contact (see Christ et al., 2010). This effect can be attributed to the fact that representatives of the ingroup provide normative information to their own group on how one should behave when interacting with members of cultures that are different from one’s own, and this would appear to reduce intergroup anxiety (Wright et al., 1997). Moreover, they can help spread the conviction that many commonly held prejudices do not correspond to reality, thus contributing to redefining the intergroup relation in less negative terms.

However, Pettigrew, Tropp, Wagner, and Christ (2011) warn regarding the change of behaviour found through indirect contact. In fact according to the authors, such change is not as strong as that found through direct contact and can change more easily.

The principle of contact theories (whether direct or indirect) applied to the set "friends of friends" is very interesting when thinking about the sibling relation in which it often happens that siblings tell each other about the vicissitudes of their relationships with friends (indirect contact - talking about friends) or invite their friends home to play or study (direct contact - becoming acquainted with the friends of one’s sibling). Both of these situations are interesting in that these friends could belong to an ethnic outgroup generally discriminated against. Nevertheless, direct and indirect contact with members of the outgroup on the part of siblings, whose dynamics are very interesting, have not been an object of interest in the literature.

## The Sibling Relation

Most of the literature on socialisation processes has mainly concentrated on “vertical” processes (that is, from parents to children) (see, for an example about prejudice, Degner & Dalege, 2013). Although parents are the principal source of influence for offspring over the entire life cycle, they are not exclusively so (Scabini, Marta, & Lanz, 2006). There is also, in fact, “horizontal” *intra*generational socialisation in which it is the peer group (friends or siblings) that acts as a partner in the exchange. The sibling relationship differentiates from the parent relationship since, while the second is mainly complementary, that of the siblings is both complementary and reciprocal (Dunn, 1983).

Siblings continue to have an important role in reach others lives into adulthood in spite of the fact that the incidence of contact diminishes after the childhood (Pulakos, 1987).

Therefore, as the siblings’ link persists through adulthood, the potential remains for siblings to have influence on one another (Cicirelli, 1991).

The sibling relationship is therefore an important context in which socialization takes place (Kavčič & Zupančič, 2005; Scabini & Iafrate, 2003). Yet this relation is unique in that siblings are a ‘social laboratory’ among peers, situated in the family where it is possible to encounter each other in a ‘horizontal’ manner by testing rules and learning the limits and boundaries that regulate and constrain one’s relationship with others.

While attention has not been focused on the influence that the siblings can have on each another regarding attitudes, thus, not on prejudice either. In the recent meta-analysis conducted by Degner and Dalege (2013), for example, the authors take into consideration exclusively the socialization of the prejudice from parents to children, not mentioning the importance also of the influence of the sibling relationship.

In the present study, we intend to analyse whether blatant and subtle prejudice in young people can be linked to contact (whether direct or indirect) experienced through the older or younger sibling’s friendships.

In light of the literature discussed above and the considerations made, our hypotheses are the following:

H1: a) *Direct contact hypothesis*: The young people whose friendship circle includes members of the outgroup (Africans) will be found to have lower levels of blatant and subtle prejudice as compared to those who do not have such friendships.

b) *Direct contact + extended contact*: The young people who, in addition to direct contact, have brothers and sisters who, in turn, have African friends will be found to have even lower prejudice values.

c) *Only extended contact*: The young people who do not have African friends but have siblings with African friends will not be found to have lower levels of prejudice as compared to the other typologies under investigation.

d) *No contact*: Finally, the young people who have neither African friends nor siblings with such friendships will be found to have higher levels of prejudice.

H2: Efficacious contact (whether direct or indirect) is contact that satisfies the requisites presented in the introductory section. Since the friendship relationship includes these requisites, we predict that only it can be linked to a significant decrease in prejudice both for the young people who have friends in the outgroup and for their siblings. Different contact typologies (direct and indirect) that do not respect the requisites called for by Allport (1954) will not produce a reduction in prejudice, either for the young people or for their siblings.

## Method

### Participants and Procedure

Data for 93 sibling dyads were collected (rate of response 93%). Of these, 5 were eliminated because the siblings were twins, and their small number did not make it possible to carry out specific analyses for this typology.

The final group of participants, therefore, consisted of 88 dyads (brothers and/or sisters), subdivided into younger brother/sister (13-29 years old, *M* = 18.78, *SD* = 3.31, 34.4% males) and older brother/sister (17-34 years old, *M* = 23.36, *SD* = 4.24, 27.2% males), for a total of 176 participants. Younger and older siblings differ in a statistically significant way with respect to age, *t*(87) = 8.37, *p* < .001. All siblings live at home with their parents and are all Italians, whose parents are both Italian.

Recruitment took place in five different departments of three Italian universities and in middle and high schools. Each enlisted person was asked to bring home an envelope containing two questionnaires and to have them also completed by a brother/sister in order to obtain complete dyads.

All participants to whom the questionnaires were distributed were informed in advance that participation was free and voluntary and that all data were to be used only for the purposes of research in an aggregate manner.

### Instruments

Each dyad was administered a questionnaire containing the following measures, in addition to questions on the socio-demographic characteristics (age, gender, etc.):

#### Subtle and blatant prejudice

We used the Subtle and Blatant Prejudice Scales (Pettigrew & Meertens, 1995) in the Italian version by Arcuri and Boca (1996). The scale is composed of two 10-item subscales, one of which measures blatant prejudice (e.g. “Italians and Africans can never feel at ease with one another, even if they become friends”) and another of which measures subtle prejudice (e.g. “It would be better if the Africans who live in our country avoided the places where their presence is not welcome”). The scale provides for a 6-step response modality (from 1 = *Absolutely in disagreement* to 6 = *Absolutely in agreement*). For reliability, see Table 1.

#### Contact frequency

4 ad hoc Items aimed at investigating contact occasion with persons of African ethnicity: “Is or was there an African among your friends?”, “Are there Africans among your neighbours?”, “Do or did you have Africans as classmates or coworkers?”, “Do or did Africans work for your family as domestic help, handymen, etc.?” with Yes/No response modalities.

The target-outgroup chosen consists of persons of African descent in that they are one of the most prevalent and longstanding ethnicities in Italy.

### Data Analysis

To test the first hypothesis, 4 different contact typologies were created: 1. Neither the older nor the younger sibling has African friends (32.9%); 2. The older as well as the younger sibling have African friends (30.7%); 3. The older sibling has African friends, but the younger one does not (18.2%); 4. The older sibling does not have African friends, but the younger one does (18.2%).

For the second hypothesis, 3 contact indicators were created (each one divided into the 4 levels mentioned above) in keeping with the logic previously presented for friendship, but with the other contact typologies taken under consideration (being neighbours, classmates or coworkers; having employees in the outgroup).

In order to analyse the differences between means for the groups, in the presence of related data such as that between siblings (Kenny, Kashy, & Cook, 2006), mixed variance analyses were conducted (Mixed ANOVA with 1 factor between and 1 factor within). The independent variable is having, or not having, friendship relationships with persons of African descent (H1) and the four typologies of contact with persons of African descent previously presented (H2); the dependent variable is the subject’s own prejudice (blatant and subtle) and that of his/her respective siblings.

## Results

Table 1 shows the response means, *SD*, Cronbach Alpha, and correlations for the scales used, divided between older and younger siblings measuring blatant and subtle prejudice.

**Table 1:** Means, SD, Cronbach α and Pearson Correlations of Blatant and Subtle Prejudice, Divided Between Older and Younger Siblings

	*M*	*SD*	1	2	3	4
Younger siblings						
1. Blatant prejudice	3.07	0.43	(.89)			
2. Subtle prejudice	3.38	0.80	.38**	(.87)		
Older siblings						
3. Blatant prejudice	2.81	1.10	.59**	.50**	(.92)	
4. Subtle prejudice	3.24	0.82	.52**	.45**	.90**	(.83)

*Note.* Coefficient alphas are presented in parantheses along the diagonal.

**p < .01.

**Figure 1:**
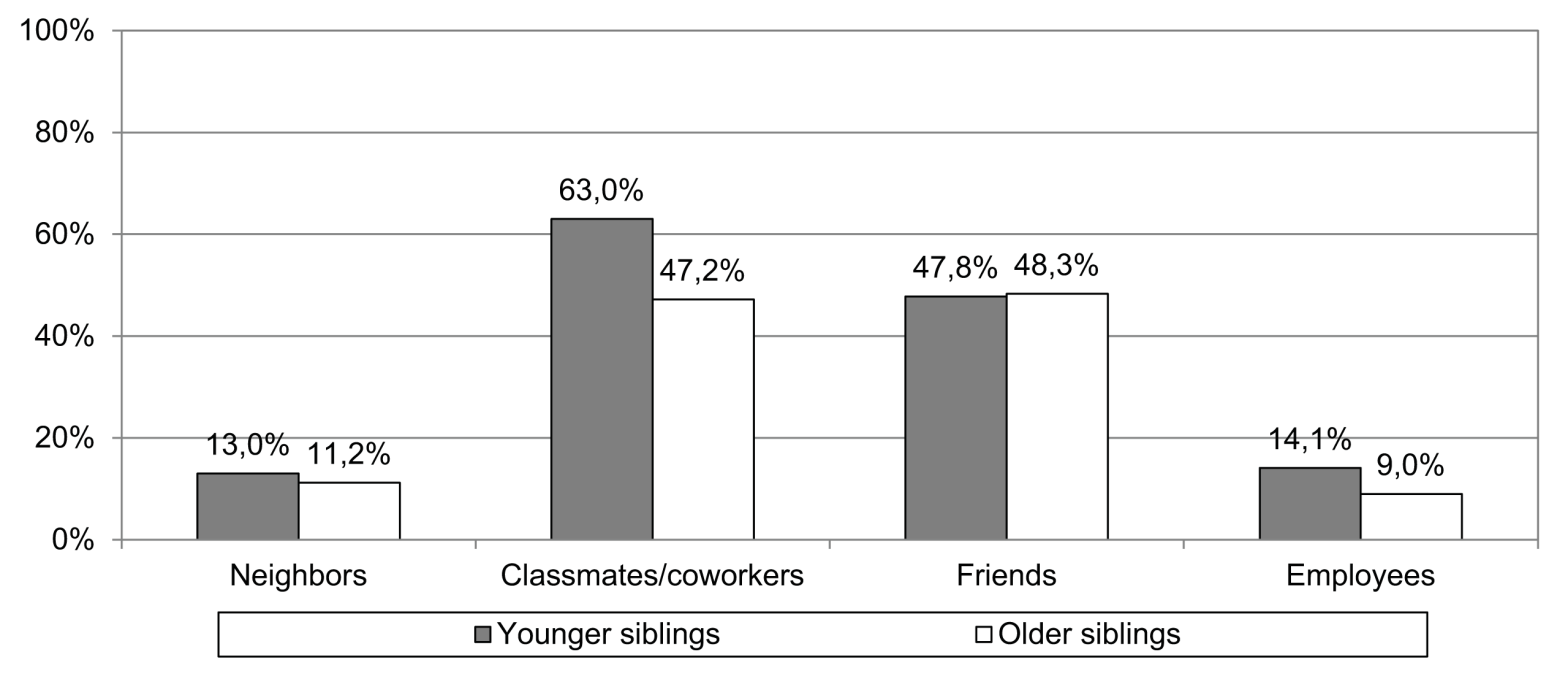
Percentages of “yes” responses regarding situations of contact and relation that siblings state that they have had with people of African descent.

As to the frequency with which siblings stated that they have (or had) contact with the outgroup, it emerged that both older and younger siblings quite frequently have had classmates or coworkers who were of African descent (respectively, 47.2% and 63.0%) in addition to having Africans as friends (48.3% and 47.8%). Less frequent were situations in which persons of African descent were either neighbours (11.2% and 13.0%) or employed by subjects’ families (9.0% and 14.1%) (Figure 1).

**Figure 2:**
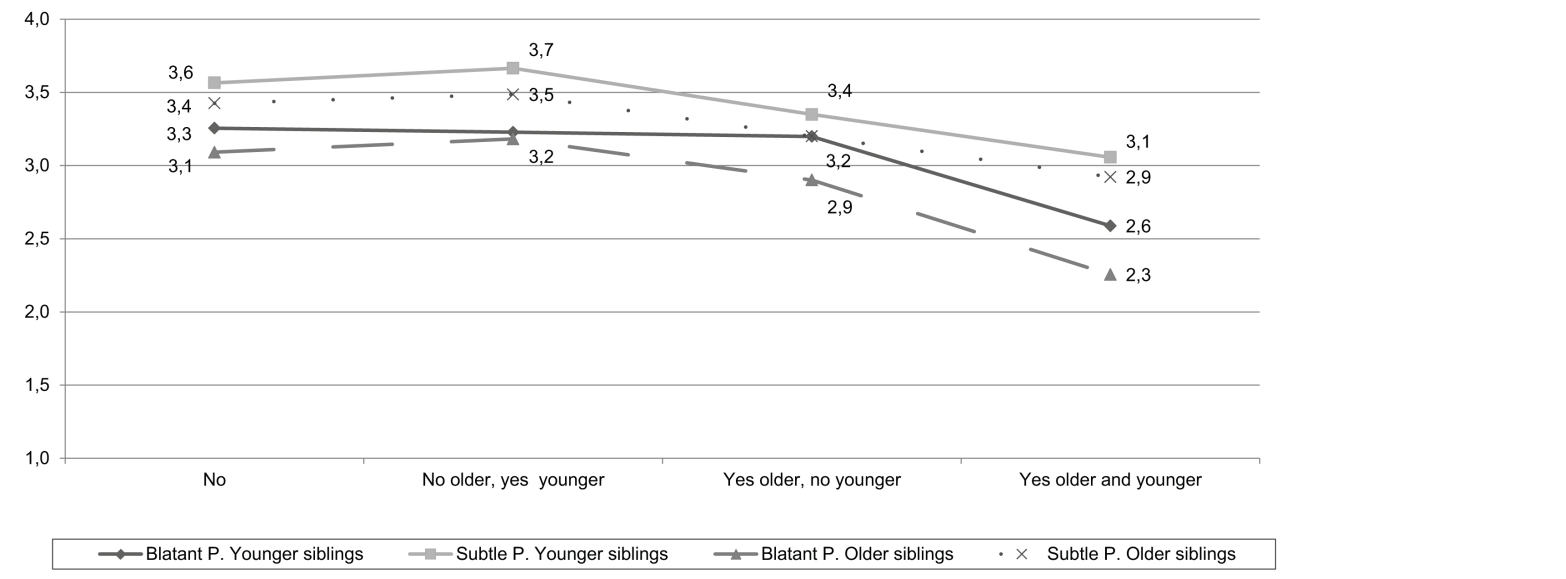
Means of siblings’ blatant and subtle prejudice with respect to four categories.

Regarding the first hypothesis, the Mixed ANOVA showed that statistically significant differences exist for subtle prejudice in the younger sibling, *F*(3, 84) = 2.69, *p* < .05, and blatant prejudice in the older sibling, *F*(3, 84) = 3.77, *p* < .05, depending on the type of contact under consideration. In particular, the post-hoc tests reveal a statistically significant difference both for subtle prejudice in the younger sibling and for blatant prejudice in the older sibling in the group of subjects who have friends in the outgroup and whose sibling also has African friends, and all the other groups (thus, both conditions must be satisfied) (Figure 2).

Regarding the second hypothesis, that is, the analysis of the potential differences in the means of blatant and subtle prejudice in both older and younger siblings with respect to the different contact typologies, it emerges that having neighbours, classmates/coworkers, or Employees does not influence any of the forms of prejudice under consideration (Table 2), nor do these typologies influence prejudice in siblings.

The only efficacious contact typology - for oneself and for one’s siblings - is thus friendship.

**Table 2:** Analyses of Variance for the Contact Typologies Investigated

	*F*	*p*
*Older siblings*		
Blatant prejudice		
Neighbors	1.17	.23
Classmates/Coworkers	0.86	.46
Having Employees	0.70	.55
Subtle prejudice		
Neighbors	1.09	.35
Classmates/Coworkers	0.73	.53
Having Employees	0.57	.63
*Younger siblings*		
Blatant prejudice		
Neighbors	1.45	.23
Classmates/Coworkers	1.13	.33
Having Employees	0.18	.90
Subtle prejudice		
Neighbors	1.18	.15
Classmates/Coworkers	1.06	.37
Having Employees	0.16	.92

*Note. df* are 3,84 for all conditions.

## Discussion

The present research set for itself the objective of testing two hypotheses. We found substantial confirmation for the first hypothesis. The post-hoc tests reveal that the older sibling reports statistically lower blatant prejudice values when not only he/she has African friends, but also his/her younger sibling has such friends. At the same time, a symmetrical but reversed situation is found for the younger sibling’s subtle prejudice, which decreases in the same conditions reported for the older sibling’s blatant prejudice, that is to say, only when he/she has African friends and when his/her sibling also has them.

These findings are of particular interest because they suggest that the more possibilities of contact and exchange (direct contact) there are, but also possibilities of having “second hand” sources (indirect contact) with respect to members of the outgroup, the more blatant prejudice, in the one case, and subtle prejudice, in the other, decrease. Furthermore, this is a very important result because the prejudice scale used does not investigate the direct relationship with specific outgroup members known personally by the subject or through a sibling, but Africans in general; therefore, we can hypothesise that this shows an effect of generalisation of a reduction of prejudice toward the entire outgroup, and not toward the individual member directly known by the subject, or about whom the subject has information.

On the contrary, for as much as concerns the indirect contact, our results confirm the doubts on the efficacy of such relationships evidenced already by Pettigrew and colleagues (2011).

The result shows that both the older sibling’s blatant prejudice as well as the younger sibling’s subtle prejudice decrease. A possible interpretation could be imputed to siblings’ roles connected to birth order: being the older sibling can cause one to feel obligated to manifest certain attitudes arising not so much from real conviction, but from what others (parents, in the first place) expect. Moreover, the older sibling may be assigned the role of “example” for the younger one, so that he/she may feel more compelled to assume socially accepted attitudes even if they are not close to his/her own ideologies or positions. Toman (1993) claimed that parents tend to have higher expectations of the older child than they do of younger siblings. As Scabini and Iafrate (2003) asserts, moreover, when there is an older sibling in a family, the parents may end up “delegating” parental care giving. Thus, it is blatant, and not subtle, prejudice that decreases. In contrast, the younger sibling could have more freedom from role expectations (as the younger sibling, he/she is not compelled to “give a good example”) so that he/she could be less constrained to adapt to implicit and explicit norms. As Kidwell (1982) asserts, younger siblings occupy a position just as unique as that of older ones in that norms and expectations are more relaxed, and parents’ attention is more focused on aspects of play and having fun (this is “the baby of the family”). This could free the younger sibling to take more personal positions and to manifest more openly his/her own attitudes.

The second hypothesis was confirmed in full, that is, that typologies of direct contact (living near people of African descent, having them as classmates or coworkers, or having them as Employees in one’s home) or indirect contact (siblings that have had these experiences), unlike friendship, do not influence blatant and subtle prejudice in young people - independently of whether the contact experience was one’s own or of one’s sibling. It turns out, in fact, that the only truly efficacious contact typology, in agreement with the literature, is one that satisfies the requisites proposed by Allport (1954) and Pettigrew and Tropp (2006).

This research also presents some points that call for further study. First of all, the number of participants considered in the present study is small, and therefore not representative of the whole country. Without a doubt, it would be of fundamental importance to expand the number of participants in order to be able to confirm the interesting results that emerged and to deepen the study, taking other variables into consideration as well. A more numerous sample would allow, for example, differentiating by gender, an aspect that was not possible to differentiate in the present work due to the limited number of males. As well, it would also have been interesting to be able to make analyses taking into consideration the age gap between the siblings, inasmuch as it is supposable that this variable could influence the relationships that are presented in this work. One last aspect in this regard concerns the fact that the data were gathered exclusively in Italy, which is a special context as concerns family relationships, as has been amply explained. It would be interesting to be able to contrast the results obtained in other countries, in order to understand whether these results are due to the context in which the present research was conducted or generalizable to other realities.

Secondly, we do not have information regarding the quality of the direct contact had by the young people, but only whether it occurred or not. This variable is of great importance: if the contact with an outgroup member did not have positive outcomes, this will influence interactions with other members of the same or other outgroups and will not decrease prejudice levels. On the contrary, it will raise them (e. g. see Prestwich, Kenworthy, Wilson, & Kwan-Tat, 2008).

In the third place, the research design utilized is merely cross-sectional. It would be of unquestionable relevance to be able to use a longitudinal design, in order to detect any changes of manifest and latent prejudice over the course of time. This is especially crucial when considering a phase of the life cycle such as the transition to adulthood, in which youth are still constructing attitudes, norms, and values (Scabini et al., 2006). Finally, it would also be interesting to investigate implicit attitudes as regards the contact experience. Often, in fact, the attitudes and opinions that the subjects profess to have do not coincide with those actually manifested or enacted in everyday situations (Arcuri & Zogmaister, 2007). Furthermore, it is possible to hypothesise that values and cognitions that are ambivalent vis-à-vis others can coexist in the individual. This ambivalence is not always reported by the subject during self-evaluation owing to social desirability and people’s limited introspective capacity.

In addition, it is opportune to remember that the data that we are analyzing are familiar, and therefore not independent of one another (Kenny, Kashy, & Cook, 2006). In this regard, it would be interesting to be able to apply other analyses that emphasize even more the dyadic nature of the sibling relationship. In this sense, future researches can take into consideration analyses of the dyadic correlation, just as done in previous researches by Alfieri and Marta (2012) and also subsequently reiterated by Degner and Dalege (2013).

### Conclusions

The present study is well suited to providing concrete suggestions for those who work in various capacities with young people and lays the groundwork for some practical considerations: in the first place, fostering opportunities for contact. Establishing contact with the outgroup helps us come to know its merits, undo stereotypes, and change our minds regarding commonly held beliefs handed down from the past. However, this concept should not be limited to “involuntary” opportunities (such as chance encounters with neighbours, classmates, or coworkers), but the preconditions must be created so that spontaneous and desired contacts take place (such as, indeed, friendship relations, which are chosen).

Secondly, siblings are a source of influence for one another, not least because they belong to the same generation, and hence resemble and influence one another, at times even more then their own parents are able to do (Alfieri, Barni, Rosnati, & Marta, 2014). Often they do not give tangible proof of understanding others’ points of view, but they “absorb” - sometimes in a critical, sometimes in a stereotypical manner - a shared feeling with respect to “others.” We should make use of this evidence in an attempt to implement interventions that are aimed not only at individuals, but are extended to the family circle, and to siblings in particular (Scabini et al., 2006).
